# Serum YKL-40 as a marker of liver fibrosis in patients with non-alcoholic fatty liver disease

**DOI:** 10.1038/srep35282

**Published:** 2016-10-14

**Authors:** Erina Kumagai, Yohei Mano, Sachiyo Yoshio, Hirotaka Shoji, Masaya Sugiyama, Masaaki Korenaga, Tsuyoshi Ishida, Taeang Arai, Norio Itokawa, Masanori Atsukawa, Hideyuki Hyogo, Kazuaki Chayama, Tomohiko Ohashi, Kiyoaki Ito, Masashi Yoneda, Takumi Kawaguchi, Takuji Torimura, Yuichi Nozaki, Sumio Watanabe, Masashi Mizokami, Tatsuya Kanto

**Affiliations:** 1 The Research Center for Hepatitis and Immunology, National Center for Global Health and Medicine, Ichikawa, Japan; 2 Department of Gastroenterology, Juntendo University School of Medicine, Hongo, Bunkyo, Tokyo, Japan; 3 Department of Pathology and Laboratory Medicine, Kohnodai Hospital, National Center for Global Health and Medicine, Ichikawa, Japan; 4 Division of Gastroenterology, Department of Internal Medicine, Nippon Medical School Chiba Hokusoh Hospital, Inzai, Japan; 5 Department of Gastroenterology and Metabolism, Hiroshima University, Hiroshima, Japan; 6 Department of Gastroenterology and Hepatology, JA Hiroshima General Hospital, Hiroshima, Japan; 7 Division of Gastroenterology, Department of Internal Medicine, Aichi Medical University School of Medicine, Nagakute, Japan; 8 Division of Gastroenterology, Department of Medicine, Kurume University School of Medicine, Kurume, Japan; 9 Department of Gastroenterology, National Center for Global Health and Medicine, Central Hospital, Tokyo, Japan

## Abstract

Non-alcoholic fatty liver disease (NAFLD) is a common cause of chronic non-viral liver disease. YKL-40, chitinase-like protein expressed in multiple tissues including liver, is involved in cell proliferation, inflammation and remodeling of the extracellular matrix. The aim of this study was to assess whether serum YKL-40 levels are associated with liver fibrosis in NAFLD patients. Serum YKL-40 levels were quantified in 111 NAFLD patients and 23 HCC patients with NAFLD. To identify the source of YKL-40, immunofluorescence staining of liver specimens from NAFLD patients was performed. Serum YKL-40 levels in NAFLD patients increased in accordance with the progression of liver fibrosis. Multivariate analysis revealed that YKL-40 was one of the independent factors significantly associated with severe fibrosis (F3-4). We established a new predictive model for fibrosis of NAFLD, using logistic regression analysis: YKL-40 based fibrosis score = −0.0545 + type IV collagen 7s * 0.3456 + YKL-40 * 0.0024. Serum YKL-40 levels of HCC patients with non-cirrhotic NAFLD were significantly higher than those without HCC. Immunofluorescence staining showed that YKL-40 was expressed by macrophages in liver tissue of NAFLD patients. In conclusion, macrophage-derived YKL-40 is a feasible biomarker of liver fibrosis in NAFLD patients.

Nonalcoholic fatty liver disease (NAFLD) is the most common cause of chronic liver disease with an estimated prevalence worldwide ranging from 25% to 45% of the entire population^1^. Annual health checkup data showed that 9–30% of Japanese adults have ultrasonography-diagnosed NAFLD^2^. Although simple steatosis is considered benign with a low risk of progression to severer liver disease, approximately 20%^3^ of NAFLD patients show histological signs of necroinflammation and fibrosis. Such pathological features indicate that those with nonalcoholic steatohepatitis (NASH) are at risk of developing cirrhosis, end-stage liver failure and hepatocellular carcinoma (HCC)^4^. Liver biopsy is considered to be the gold standard for distinguishing simple steatosis from NASH and for staging fibrosis. Longitudinal studies of histological features in NAFLD patients have shown that fibrosis is the best independent predictor, followed by portal inflammation, and ballooning^5^. However, liver biopsy has several limitations in the management of NAFLD patients. Because of its invasiveness and risk of complications, liver biopsy cannot be easily applied to clinical practice. In addition, sampling bias has been reported in patients with NAFLD and might affect both diagnosis and staging of the disease. Given these limitations, a number of noninvasive markers including both serologic and imaging methods have been evaluated for NAFLD patients to estimate steatosis, NASH, and fibrosis.

YKL-40 (or chitinase 3-like-1, breast regression protein-39 or human cartilage glycoprotein-39) is a chitinase-like protein which is found in humans as a secretion product of articular chondrocytes and synovial cells^6^. YKL-40 lacks chitinase enzyme activity due to mutations within the active site^7^. Serum YKL-40 levels are elevated in patients with a wide array of human diseases characterized by sustained inflammation, including rheumatoid arthritis, atherosclerosis, type 2 diabetes, chronic pancreatitis, asthma, and Crohn’s disease^8^,^9^,^10^,^11^. YKL-40 is expressed by various cell types, including neutrophils^12^, macrophages^13^ and several types of malignant tumors^14^. The serum YKL-40 level has been evaluated as a noninvasive marker of various chronic inflammatory and fibrotic liver diseases, including chronic hepatitis C (CHC)^15^ chronic hepatitis B (CHB)^16^ and alcoholic liver disease^17^. Secreted by activated macrophages, YKL-40 is believed to act as a chemoattractant for endothelial cells, can modulate angiogenesis during tissue repair and is expressed in multiple tissues including human liver^18^,^19^,^20^.

In this study, we evaluated the feasibility of YKL-40 as a diagnostic marker of fibrosis in patients with NAFLD. We also identified the cells capable of expressing YKL-40 in the liver of NAFLD.

## Results

### Serum YKL-40 levels in NAFLD patients increased with the progression of fibrosis

**Figure 1:**
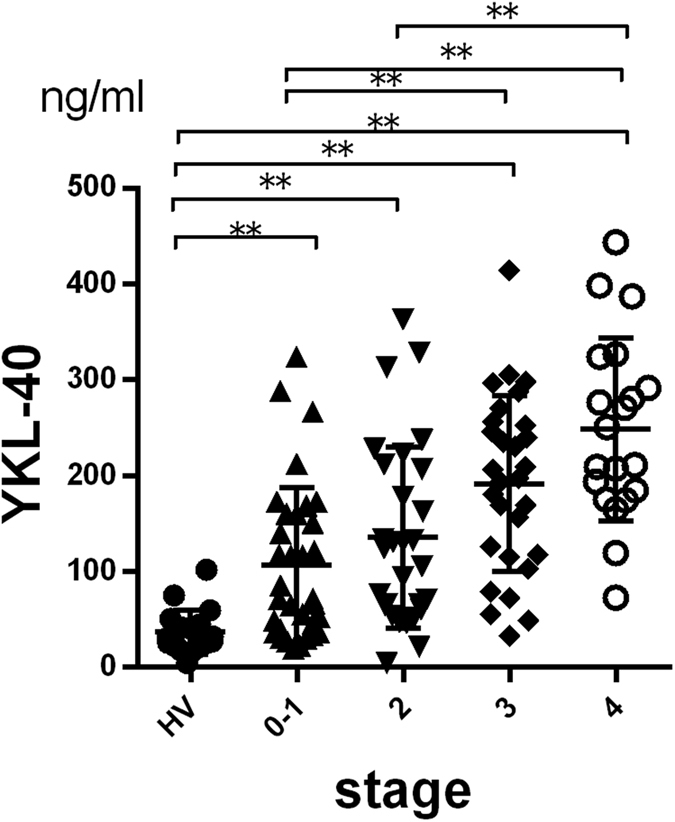
Serum YKL-40 levels in patients with NAFLD. Serum YKL-40 levels in NAFLD patients increased with the progression of fibrosis (Brunt classification). *p < 0.05, **p < 0.001, by Kruskal-Wallis test with Dunn’s multiple comparison test. HV, healthy volunteers.

**Figure 2:**
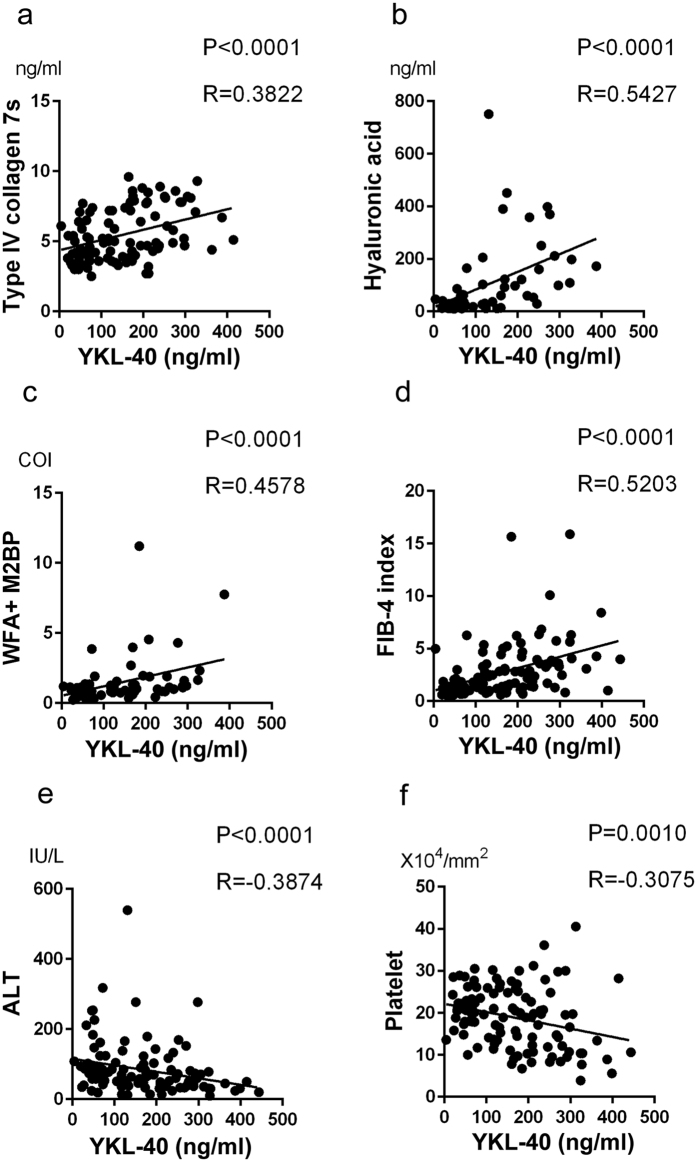
Serum YKL-40 levels correlated with other biomarkers and fibrosis scores in patents with NAFLD. The correlations between YKL-40 and each marker (**a**) type IV collagen 7s; (**b**) hyaluronic acid; (**c**) WFA^+^ -M2BP; E, ALT and F, platelet) and fibrosis score (**d**): FIB4-index) were assessed by Spearman’s rank-correlation coefficient. The p-values and correlation coefficients are shown in each plot.

First, to assess the feasibility of YKL-40 as a biomarker of fibrosis in NAFLD patients (Table 1), we measured serum YKL-40 levels and other fibrosis markers such as type IV collagen 7s, hyaluronic acid, WFA^+^ -M2BP and clinically accepted fibrosis scores (AST-to-platelet ratio index (APRI) and FIB4-index). In parallel with the progression of fibrosis in NAFLD patients, serum YKL-40 levels increased, showing a difference among the groups of HV or stages 0-1 and those of stages 3 or 4 (Fig. 1). Similar results were obtained with type IV collagen 7s, hyaluronic acid, WFA^+^ -M2BP, APRI, and FIB-4 index (Supplementary Figures S1a–e). Regarding the correlation among such factors, YKL-40 levels were positively correlated with type IV collagen 7s, hyaluronic acid, FIB-4 index, and WFA^+^ -M2BP, while they were inversely correlated with ALT and platelet count (Fig. 2a–f). In 65 patients whose histological activity score were available (Supplementary Table S1), serum YKL-40 levels did not correlate with any scores of steatosis, inflammation and ballooning (data not shown). We also examined serum YKL-40 levels in CHC patients (Supplementary Table S2). In parallel with the progression of fibrosis in CHC patients, serum YKL-40 levels increased (Supplementary Figure S2).

### Serum YKL-40 showed a significant association with liver fibrosis in NAFLD patients

To determine the ability to diagnose fibrosis stage, we divided all cases into two groups, such as moderate fibrosis (stage 0–2) and severe fibrosis (stage 3-4). We performed univariate analysis on age, BMI, platelet, albumin, AST, ALT, YKL-40, type IV collagen 7s, hyaluronic acid, WFA^+^ -M2BP, APRI, and FIB-4 index. Univariate analysis revealed that YKL-40, type IV collagen 7s, hyaluronic acid, APRI, and FIB4-index are associated with classification for severe fibrosis (Table 2). By multivariate analysis, YKL-40 [odds ratio (OR) 1.007, 95% confidence interval (CI) 1.000–1.014, P = 0.0412] and type IV collagen 7s [OR 1.974, 95% CI 1.316–2.962, P = 0.0010] were independent factors for severe fibrosis (Table 2).

### A diagnosis model was developed for NAFLD patients with severe fibrosis using YKL-40 and type IV collagen 7s

Next, we evaluated serum YKL-40 as a diagnostic marker for classification of severe fibrosis. By using ROC analyses for classification of severe fibrosis in NAFLD patients, we obtained the cutoff values of serum YKL-40 (165 ng/mL), type IV collagen 7s (6.1 ng/ml), hyaluronic acid (46 ng/ml), WFA^+^ -M2BP (1.12 COI), APRI (0.89), and FIB-4 index (1.6) (Table 3, Supplementary Figure S3). The best ROC result from serum fibrosis markers was applied by measuring the area under the curve (AUC), with type IV collagen 7s showing the highest AUC (0.8458). The AUC of YKL-40 was 0.7683, being superior to that of hyaluronic acid (0.7527), WFA^+^ -M2BP (0.6953), and APRI (0.7429). Because YKL-40 and type IV collagen 7s were independent factors for classification of severe fibrosis, we performed logistic regression analysis and established the following new predictive model for severe fibrosis in NAFLD patients:


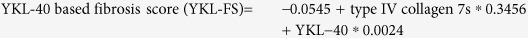


The cutoff value for severe fibrosis in this model was 2.06. The AUC of YKL-FS (0.8763) were higher than that of YKL-40 (0.7638) and type IV collagen 7s (0.8458) alone (Table 3).

In order to assess the feasibility of YKL-40 and YKL-FS as a diagnostic marker for other fibrosis stages, we examined these 2 parameters for the classification of advanced fibrosis (F ≥ 2) and liver cirrhosis (F = 4). The AUC of YKL-40 alone was 0.7380 (F ≥ 2) and 0.7392 (F = 4), and the AUC of YKL-FS was 0.8513 (F ≥ 2) and 0.8998 (F = 4). Therefore, the diagnostic accuracy of YKL-40 and YKL-FS in stages of F ≥ 2 and F = 4 were comparable with that in F ≥ 3.

### YKL-40 was expressed by macrophages in NAFLD liver tissue and *in vitro*

**Figure 3:**
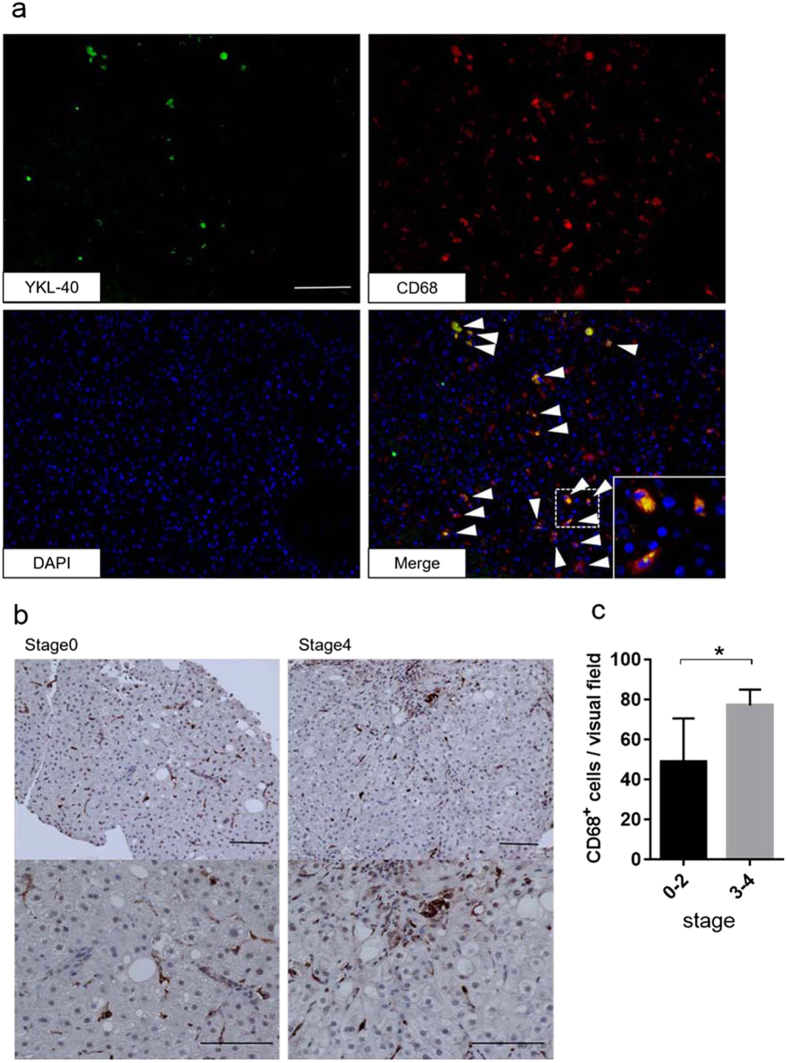
Immunofluorescence and immunohistochemical staining of YKL-40 and CD68+ cells in liver tissue from NAFLD patients. (**a**) Immunofluorescence staining on frozen liver biopsy specimen from patients with NAFLD. (x200, green, YKL-40; red, CD68; blue, DAPI). White arrowheads mark the positions of YKL-40 and CD68 positive cells. Inset of the photomicrograph shows fluorescent merged cells in enlarged scale. Scale bar represents 100 μm. (**b**) CD68 staining on paraffin embedded specimens from NAFLD patients (stage 0, left section; stage 4, right section; x200, upper section; x400, lower section). Scale bars represent 100 μm. (**c**): The number of CD68 positive cells per visual field is shown by the stage of fibrosis. CD68 positive cells accumulate according to the severity of the liver fibrosis. Five paraffin sections were evaluated. *p < 0.05 by Student t-test.

Immunofluorescence staining of YKL-40 and CD68 in liver tissue from NAFLD patients showed that several CD68+ cells expressed YKL-40 (Fig. 3a). This finding suggests that macrophages are likely to secrete YKL-40 in chronic liver disease. In addition, we immunohistochemically stained NAFLD liver section for CD68, as CD68 positive cells significantly increased with the progression of liver fibrosis (Fig. 3b,c). A larger number of macrophages accumulated in fibrotic areas compared to non-fibrotic areas. An increased number of CD68+ cells in the liver of severe fibrosis may be involved in higher levels of serum YKL in such patients. In order to confirm that YKL-40 is a macrophage-derived factor, we evaluated YKL-40 production by cultured primary human macrophages *in vitro*. After 5–7 days in culture, the levels of YKL-40 transcription increased over time (Supplementary Figure S4a). Next, we stimulated macrophages with IL-1β and/or TNFα, which have been reported to be upregulated in NAFLD or severely obese patients^21^,^22^. Interestingly, YKL-40 levels were markedly increased with TNF-α or the combination of TNFα and IL-1β (Supplementary Figure S4b). These results suggest that the increased levels of IL-1β and TNFα are associated with the increase of serum YKL-40 in NAFLD patients. However, in this study, there was no correlation between serum levels of YKL-40 and TNFα or IL-1β (Supplementary Figure S5), suggesting that serum YKL-40 levels are determined not only by other cytokines but also the number of macrophages.

### Serum YKL-40 levels were elevated in NAFLD patients with HCC

**Figure 4:**
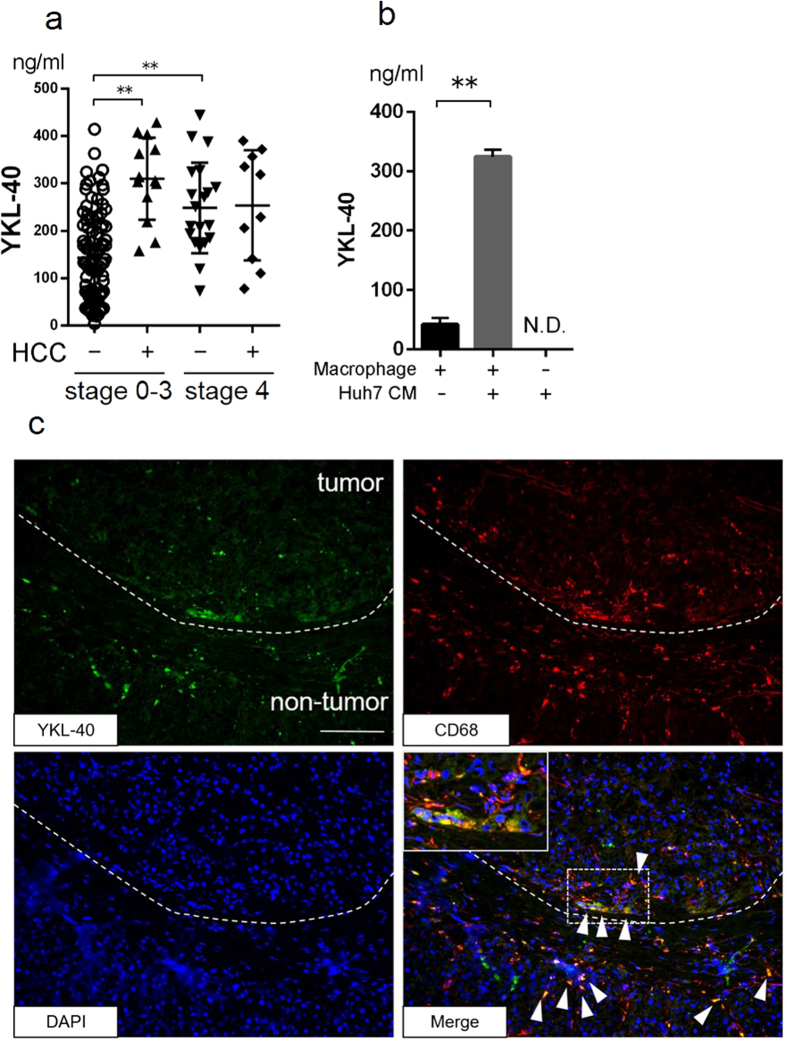
Serum YKL-40 levels and YKL-40 expression in NAFLD patients with HCC. (**a**) Serum YKL-40 levels in fibrosis stage 0–3 and stage 4 patients with or without HCC are shown.*p < 0.05, **p < 0.001 by Kruskal-Wallis test with Dunn’s multiple comparison test. (**b**) YKL-40 levels in culture medium of macrophage treated with DMEM or the conditioned medium of Huh7 are shown. The YKL-40 level in the Huh7 conditioned medium is shown. *p < 0.05, **p < 0.001, by Student t-test. N.D., not detected. (**c**) Immunofluorescence staining of YKL-40 and CD68 in frozen liver specimens from NAFLD patients with HCC. The dotted line indicates the margin between tumor and non-tumor. White arrowheads mark the positions of YKL-40 and CD68 positive cells. The inset of the photomicrograph shows fluorescent merged cells in enlarged scale. Scale bar represents 100 μm. (x200, green, YKL-40; red, CD68; blue, DAPI). CM, conditioned medium; DMEM, Dulbecco’s Modified Eagle’s Medium, N.D., not detected.

In order to examine whether serum YKL-40 levels are associated with the presence of HCC, we compared serum YKL-40 levels between NAFLD patients with HCC and those without. In NAFLD patients (F0–F3) with HCC, serum YKL-40 levels were significantly higher than in those without HCC (mean 309.5 ± 86.7, 143.2 ± 94.8 respectively, P < 0.0001) (Fig. 4a). On the other hand, in cirrhotic NAFLD patients (LC, F4) with HCC, serum YKL-40 levels were not significantly different from those without HCC (Fig. 4a). Also in CHC patients, serum YKL-40 levels were significantly higher in those with HCC than in those without HCC (Supplementary Figure S6). In cirrhotic patients with HCV infection, YKL-40 levels did not differ between those with and without HCC (Supplementary Figure S6). To investigate whether YKL is secreted from macrophages in the presence of HCC, cultured macrophages at day 5 were treated with the conditioned medium (CM) of Huh7 (human HCC cell line). YKL-40 levels released from macrophages with addition of CM were significantly increased compared to that without CM. YKL-40 was not measurable in the Huh7 CM (Fig. 4b). These results suggest that certain HCC-derived humoral factors could stimulate macrophages into releasing YKL-40.

### YKL-40 was expressed in macrophages surrounding HCC

We performed immunofluorescence staining of YKL-40 and CD68 in frozen sections of liver tissue from NAFLD patients with HCC. We found that macrophages located in the peritumoral area of HCC expressed YKL-40. Some of the YKL-40 positive macrophages were also identified in fibrosis areas and sinusoidal areas in the liver (Fig. 4c).

## Discussion

The present study showed that serum YKL-40 levels are increased in NAFLD patients with fibrosis. Based on these findings, we propose a new diagnostic for severe fibrosis based on a regression equation including YKL-40 and type IV collagen 7s.

The cellular source of YKL-40 in the liver has been controversial. Johansen JS *et al*. reported that hepatic stellate cells are the main source of YKL-40^23^. In contrast, other previous studies^24^,^25^ and our examination failed to show that LX2 (human hepatic stellate cell line) and primary fibroblasts secrete YKL-40 (data not shown). Alternatively, it is reported that activated macrophages secrete YKL-40^13^,^26^,^27^. In this study, immunofluorescence staining revealed that macrophages, but not other cells, in the liver tissue express YKL-40. Support for this comes from our finding that primary human macrophages secreted YKL-40 *in vitro*. Macrophages are known to increase in parallel with the progression of liver fibrosis^28^. In this study, more macrophages were also infiltrated in the liver tissue with severe fibrosis than those of mild fibrosis (Fig. 3c). Therefore, it is plausible that the increase of activated macrophages in the fibrous liver enhances serum YKL-40 levels in NAFLD patients with severe fibrosis.

A positive correlation between YKL-40 and liver fibrosis has been reported elsewhere in patients with other causes of liver disease. Also in this study, serum YKL-40 levels are associated with fibrosis stage in patients with HCV infection (Supplementary Figure S2). These findings raise the possibility that, regardless of etiology of liver disease, YKL-40 could promote fibrogenesis; otherwise it is simply a co-existing factor with liver fibrosis. Rehli *et al*. reported the YKL-40 was regarded as a macrophage differentiation marker^13^,^29^. Therefore, the parallel increase of YKL-40 with liver fibrosis implies that the graded macrophage differentiation, as reflected by YKL-40 levels, plays key roles in liver fibrosis in NAFLD.

The present study showed that YKL-40 secretion by macrophages is upregulated by TNFα and IL-1β *in vitro*. TNFα and IL-1β are proinflammatory cytokines involved in the pathogenesis of NAFLD. Recklies *et al*. also reported that these two cytokines could stimulate YKL-40 expression in chondrocytes^30^. TNFα has a role in the development of every aspect of NAFLD (steatosis, necrosis, apoptosis and fibrosis) as well as insulin resistance^31^,^32^,^33^. IL-1β has been shown to have a role in the transformation of steatosis to steatohepatitis and liver fibrosis^34^. Thus, it is conceivable that YKL-40 is induced by macrophages stimulated by locally-abundant proinflammatory cytokines. We found that serum levels of TNFα or IL-1β were significantly higher in NAFLD patients compared with that in healthy volunteers (data not shown). However, there was no correlation with serum YKL-40 levels and serum TNFα or IL-1β levels (Supplementary Figure S5). Serum YKL-40 levels might be influenced not only cytokine stimulation but also the number of macrophages.

Higher levels of YKL-40 in HCC patients with NAFLD were observed only in cases without LC. One of the possible explanations for this is that serum levels of YKL-40 are influenced more by the fibrotic change in the liver, which may surpass the positive impact on YKL-40 by HCC. Pan J. J. *et al*. reported that the expression of YKL-40 was higher in clinical HCC specimens than in non-cancerous liver tissue^24^. In contrast, in this study, we showed that peritumoral macrophages, instead of HCC cells, expressed YKL-40 (Fig. 4c). One of the plausible reasons of such discordant results is the difference in the etiology of hepatitis or the grade of HCC between the studies. Previous studies were performed with patients having viral hepatitis and more advanced grades of HCC, which differed from our study.

The limitation of our study is that the biological function of YKL-40 in the process of liver fibrosis has not been clarified. Several reports showed that YKL-40 has substantial roles in cell proliferation and differentiation, angiogenesis, inflammation, remodeling of the extracellular matrix, and innate immune response^23^,^35^,^36^,^37^. Therefore, YKL-40 may have some important, but undetermined roles in fibrogenesis of NAFLD.

In summary, serum YKL-40 is a feasible macrophage-derived biomarker reflecting liver fibrosis in patients with NAFLD.

## Materials and Methods

### Subjects

In this study, we enrolled 111 NAFLD patients and 23 HCC patients with NAFLD, who were followed at the National Center for Global Health and Medicine, Kohnodai Hospital, JA Hiroshima General Hospital, Nippon Medical School Chiba Hokusoh Hospital, Kurume University Hospital, National Center for Global Health and Medicine, Central Hospital, and Aichi Medical University Hospital. The clinical backgrounds of these patients are shown in Table 1. The diagnosis of NAFLD was based on a liver biopsy specimen showing steatosis (≥5% of hepatocytes containing fat droplets) and the exclusion of other causes of liver disease, such as viral hepatitis, alcoholic liver disease (quantity of ethanol intake > 20 g/day for women and 30 g/day for men), drug-induced or autoimmune liver diseases. No concomitant diseases or conditions causing secondary steatohepatitis, such as endocrine disorders, primary dyslipidemia or malnutrition, were confirmed in the subjects. As disease controls, we enrolled 48 CHC patients and 31 HCC patients with CHC who were followed at the National Center for Global Health and Medicine, Kohnodai Hospital (Supplementary Table S1). We also examined 19 healthy volunteers (HV) who were negative for HBsAg, anti-HCV and anti-human immunodeficiency virus antibody/antigen and had no apparent history of liver disease or malignancies. The study was approved by the ethics committee in the National Center for Global Health and Medicine, and all methods were performed in accordance with the relevant guidelines and regulations. Written informed consent was obtained from all patients at their enrollment.

### Histological diagnosis

All patients enrolled in this study underwent liver biopsy or surgical resection for the diagnosis or treatment of HCC. For the staging of NAFLD, expert pathologists in each facility evaluated tissue fibrosis according to Brunt’s criteria^38^ and NAFLD Activity Score^39^. The staging of the disease in patients with CHC was based on the METAVIR score^40^.

### Biochemical analysis and calculation of fibrosis indices

Serum samples were collected from all patients before liver biopsy or surgical resection. The biochemical variables were quantified using standard assays and methods at the respective hospitals. APRI and FIB-4 index were calculated as previously reported^41^,^42^.

### Quantification of YKL-40 and WFA^+^ -M2BP

YKL-40 levels in serum and cell culture medium were quantified by using the YKL-40 enzyme-linked immunosorbent assay kit (Quidel, San Diego, CA) according to the manufacturer’s protocol. WFA^+^ -M2BP was quantified by WFA antibody immunoassay using a chemiluminescence enzyme immunoassay machine (HISCL-5000; Sysmex, Kobe, Japan) as previously reported^43^.

### Immunofluorescence and immunohistochemistry staining

Tissue specimens were obtained from liver biopsy sections of the NAFLD patients. YKL-40 and CD68 expression in frozen sections were observed using immunofluorescence. The primary antibodies, polyclonal goat-anti-human YKL-40 (R&D Systems, Minneapolis, MN, 1:10) and monoclonal mouse-anti-human CD68 (Novus Biologicals, Littleton, CO, 1:100), were incubated together for 90 min at room temperature. Alexa Fluor 488 donkey-anti-goat (Abcam, Cambridge, UK, 1:500) and Alexa Fluor 594 goat-anti-mouse (InvitroGen, San Diego, CA, 1:1000) were incubated in the dark for 60 min at room temperature. The sections were covered with Vectashield with DAPI (Nacalai Tesque, Kyoto, Japan). Photographs were taken with a confocal microscope.

Paraffin sections of liver from the patients were stained with monoclonal mouse-anti-human CD68 by VECTASTATIN Elite ABC kit (Vector Laboratories, Burlingame, CA) according to the manufacturer’s protocol. Five visual-fields per slide were analyzed at 400x magnification by an investigator blind to the specimen background. Visual-fields were selected randomly and contained two Glisson capsule areas in each case.

### Primary human macrophage cell culture

We collected blood from healthy volunteers and processed them for PBMCs. Monocytes were isolated from PBMCs by the MACS system, using anti-CD14 microbeads (Miltenyi Biotec, Auburn, CA) according to the manufacturer’s protocol. Monocytes were cultured for 4 days in Dulbecco’s Modified Eagle’s Medium with high glucose (DMEM, Wako, Osaka, Japan) in 24 well plates (1.0 × 10^6^/well) at 37 °C in 5% CO_2_ atmosphere with M-CSF (50 ng/ml) to obtain differentiated macrophages. At day 4 of culture, the medium was replaced with M-CSF free DMEM.

The RNA of macrophages at 5–7 days of culture was extracted for measurement of YKL-40 transcripts. RNA was extracted from the cells using ISOGEN (Nippon Gene, Toyama, Japan). cDNA was generated using transcriptor Universal cDNA Master (Roche, Mannheim, Germany). Transcripts were measured using TaqMan probes for YKL-40 (chitinase 3-like1, Hs01072228), and GAPDH (Hs2758991) with TaqMan Gene Expression Master Mix (Applied Biosystems, Carlsbad, CA), and subsequently analyzed on a Light Cycler 480 II (Roche, Germany). All data shown were from a single experiment using triplicate samples.

Differentiated macrophages at day 5 in culture were stimulated with pro-inflammatory stimuli TNFα (10 ng/ml), IL-1β (25 ng/ml), TNFα (10 ng/ml) and IL-1β (25 ng/ml), and CM of Huh7. Huh7 cells were cultured in 24-well plates (2.5 × 10^4^/well) with DMEM, and after 24 h the medium was replaced with fetal bovine serum (FBS, GE Healthcare Bio-Sciences, Pittsburgh, PA) free DMEM, then the CM of Huh7 cells was collected after 48 h of culture. Macrophage culture medium was collected at day 7 of culture. YKL-40 ELISA was performed on the macrophage culture medium. All DMEM for cell culture contained 10% FBS, 100 U/ml penicillin and 100 μg/ml streptomycin (Nacalai Tesque). M-CSF and TNFα were purchased from R & D Systems. IL-1β was purchased from Peprotech (Rocky Hill, NJ).

### Statistical analysis

The differences between two groups were assessed by Mann-Whitney U-test and Student t-test. For the analyses of groups, Kruskal-Wallis and Dunn’s multiple tests were used. Associations among the variables were determined by the χ^2^ method or Fisher’s exact test. Univariate and multivariate analyses were performed by the logistic regression model. Possible subsets of candidate variables were examined using receiver-operating characteristic (ROC) analyses for moderate fibrosis (Stage 0–2) or severe fibrosis (Stage 3-4). Sensitivity, specificity, positive and negative predictive values (PPV and NPV), and predictive accuracy were determined for appropriate cutoff values based on the ROC curves. Cochran-Armitage’s trend test was used for the categorical data analysis. A P value of less than 0.05 was considered statistically significant. Statistical analyses were performed with Graph Pad Prism software (version 6; Graph Pad Prism, San Diego, CA), SPSS statistical software (version 22.0; SPSS, Chicago, IL) and Microsoft Excel (version 2015) where appropriate.

## Additional Information

**How to cite this article**: Kumagai, E. *et al*. Serum YKL-40 as a marker of liver fibrosis in patients with non-alcoholic fatty liver disease. *Sci. Rep.* **6**, 35282; doi: 10.1038/srep35282 (2016).

## Supplementary Material

Supplementary Information

## Figures and Tables

**Table 1:** Clinical and Serological Background of Subjects.

NAFLD						
	Stage 0-1 (n = 33)	Stage 2 (n = 29)	Stage 3 (n = 29)	Stage 4 (n = 20)	HCC without LC (n = 13)	HCC with LC (n = 10)
Male/Female	16/17	12/17	8/21	6/14	10/3	7/3
Age (year)	55 (25–78)	60 (19–81)	64 (31–83)	68 (20–51)	74.3 (56–91)	74.6 (66–82)
BMI (kg/m^2^)	28.8 (22.2–38.5)	27.1 (20.8–37.4)	28.0 (24.7–34.9)	28.0 (20.1–36.9)	24.6 (18.3–31.29)	23.7 (20.9–26.6)
Platelet (x10^4^/mm^3^)	20.5 (7.8–30)	20.6 (10.4–40.5)	19.7 (9.5–30.5)	9.6 (3.9–25.1)	16.5 (7.9–24.8)	12.4 (2.9–27.1)
Albumin (g/dL)	4.3 (3.2–5.3)	4.4 (3.6–5.4)	4.3 (3.4–4.9)	4.0 (2.6–4.7)	3.9 (3.3–4.8)	3.9 (2.9–4.5)
Total bilirubin (mg/dl)	0.7 (0.3–2.6)	0.8 (0.3–2.4)	1.0 (0.4–2.4)	1.2 (0.3–2.5)	1.2 (0.6–1.3)	0.9 (0.5–3.9)
AST (IU/U)	40 (15–147)	43 (17–245)	67 (26–186)	53 (26–120)	59 (17–256)	48 (21–84)
ALT (IU/L)	77 (14–277)	74 (12–29)	71 (32–318)	42 (10–138)	43 (15–114)	44 (13–69)
Type IV collagen 7s (ng/ml)	3.9 (2.7–7.1)	5.0 (2.5–9.3)	6.2 (3.8–8.9)	7.8 (5.8–9.6)	7.0 (5.0–9.0)	9.9 (7.0–14.0)
Hyaluronic acid (ng/ml)	27 (10–33)	43 (12–751)	65 (12–267)	167 (29–451)	144 (25–218)	386 (70–1380)
WFA^+^-M2BP (COI)	0.80 (0.24–3.87)	0.74 (0.3–2.32)	0.84 (0.3–5.08)	1.95 (1.13–11.2)		
APRI	0.53 (0.23–2.36)	0.67 (0.25–2.86)	1.19 (0.33–3.41)	1.53 (0.45–3.57)	1.00 (0.18–2.58)	1.73 (0.51–4.14)
FIB-4 index	1.27 (0.46–5.64)	1.40 (0.55–4.98)	2.76 (0.70–6.83)	5.12 (1.27–15.89)	4.00 (1.52–11.09)	7.17 (2.63–15.04)
AFP (ng/ml)					196 (2.1–1241)	4.6 (0.9–8.1)
PIVKA-II (mAU/ml)					4367 (18–22448)	369.8 (10–3205)

Data are presented as the median (range).

BMI, body mass index; APRI, AST-to-platelet ratio index.

**Table 2:** Variable Parameters Associated With Advanced Fibrosis (F3 + F4) According to Univariate and Multivariate Analyses.

	Advanced Fibrosis (F3 + F4)					
	UVA			MVA		
	OR	95% CI	P-value	OR	95% CI	P-value
Age	**1.040**	**1.006**–**1.075**	**0.0206**	1.018	0.969–1.070	0.4700
BMI	1.014	0.910–1.131	0.7981			
Platelet	**0.936**	**0.878**–**0.999**	**0.0476**	0.981	0.888–1.084	0.7059
Albumin	0.456	0.166–1.257	0.1292			
AST	**1.014**	**1.002**–**1.026**	**0.0216**	1.007	0.993–1.022	0.3273
ALT	1.000	0.995–1.005	0.9678			
YKL-40	**1.010**	**1.004**–**1.017**	**0.0013**	**1.007**	**1.000**–**1.014**	**0.0412**
Type IV collagen 7s	**2.505**	**1.613**–**3.889**	**<0.0001**	**1.974**	**1.316**–**2.962**	**0.0010**
Hyaluronic acid	**1.017**	**1.004**–**1.030**	**0.0092**	0.997	0.991–1.003	0.3496
WFA^+^-M2BP	**2.516**	**1.312**–**4.828**	**0.0055**	1.190	0.604–2.345	0.6146
APRI	**3.460**	**1.720**–**6.959**	**0.0005**			
FIB-4-index	**1.937**	**1.388**–**0.066**	**0.0001**			

UVA, univariate analysis; MVA, multivariate analysis; OR, odds ratio; CI, confidence interval.

BMI, APRI, see Table 1.

**Table 3:** Performance of YKL-40 and Other Biomarkers to Diagnose Advanced Fibrosis by ROC Analyses.

	YKL-40	Type IV collagen 7s	Hyaluronic acid	WFA^+^-M2BP	APRI	FIB4-index	YKL-40 and Type IV collagen 7s
AUC	0.7638	0.8458	0.7527	0.6953	0.7429	0.7853	0.8763
Cutoff value	165.0	6.1	46	1.12	0.89	1.60	2.06
Sensitivity (%)	70.0	67.5	80.0	57.5	75.0	77.5	85.0
Specificity (%)	76.8	83.9	69.6	75.0	67.9	67.9	78.6
PPV (%)	68.3	75.0	65.3	62.2	62.5	63.3	73.9
NPV (%)	78.2	78.3	83.0	71.2	79.2	80.9	88.0
Predictive accuracy (%)	74.0	77.1	73.9	67.7	70.9	71.9	81.3

The optimal cutoff values are determined as those yielding the minimal value for (1 − sensitivity)^2^ + (1 − specificity)^2^. Such values are the closest to the (0, 1) point on the receiver-operating characteristic (ROC). AUC, area under receiver operating characteristic; PPV, positive predictive values; NPV, negative predictive values.

APRI, see Table 1.
