# Supplementary material for: Serum YKL-40 as a marker of liver fibrosis in patients with non-alcoholic fatty liver disease
Source: Sci Rep. 2016 Oct 14;6:35282. doi: 10.1038/srep35282 (PMC5064386; doi:10.1038/srep35282)

Supplementary Information

Serum YKL-40 as a marker of liver fibrosis in patients with non-alcoholic fatty liver disease

Erina Kumagai[1,2,#], Yohei Mano[1,#], Sachiyo Yoshio[1], Hirotaka Shoji[1], Masaya Sugiyama[1], Masaaki Korenaga[1], Tsuyoshi Ishida[3], Taeang Arai[4], Norio Itokawa[4], Masanori Atsukawa[4], Hideyuki Hyogo[5,6], Kazuaki Chayama[5], Tomohiko Ohashi[7], Kiyoaki Ito[7], Masashi Yoneda[7], Takumi Kawaguchi[8], Takuji Torimura[8], Yuichi Nozaki[9], Sumio Watanabe[2], Masashi Mizokami[1] and Tatsuya Kanto[1]

1 The Research Center for Hepatitis and Immunology, National Center for Global Health and Medicine, Ichikawa, Japan.

2 Department of Gastroenterology, Juntendo University School of Medicine, Hongo, Bunkyo, Tokyo, Japan.

3 Department of Pathology and Laboratory Medicine, Kohnodai Hospital, National Center for Global Health and Medicine, Ichikawa, Japan.

4 Division of Gastroenterology, Department of Internal Medicine, Nippon Medical

School Chiba Hokusoh Hospital, Inzai, Japan.

5 Department of Gastroenterology and Metabolism, Hiroshima University, Hiroshima, Japan.

6 Department of Gastroenterology and Hepatology, JA Hiroshima General Hospital, Hiroshima, Japan.

7 Division of Gastroenterology, Department of Internal Medicine, Aichi Medical University School of Medicine, Nagakute, Japan.

8 Division of Gastroenterology, Department of Medicine, Kurume University School of Medicine, Kurume, Japan.

9 Department of Gastroenterology, National Center for Global Health and Medicine, Central Hospital, Tokyo, Japan.

# Erina Kumagai and Yohei Mano contributed equally to this work.

Corresponding author: Tatsuya Kanto, M.D., Ph.D.

The Research Center for Hepatitis and Immunology, National Center for Global Health and Medicine, 1-7-1, Kohnodai, Ichikawa 272-8516, Japan.

E-mail: kantot@hospk.ncgm.go.jp

Telephone: +81-47-372-3501, Fax: +81-47-375-4766

Supplementary Table S1. NAFLD Activity Score (NAS) and Background of Patients in Each Stage of Fibrosis

NAS	Stage 0-1 (n = 12)	Stage 2 (n= 21)	Stage3 (n = 23)	Stage4 (n=9)
Male/Female	4/8	8/13	7/16	4/5
Age (year)	61 (32-74)	59 (22-81)	61 (31-79)	67 (56-80)
BMI (kg/m²)	28.6 (22.8-30.4)	28.6 (20.8-35.4)	28.2 (24.7-33.2)	27.2 (20.1-29.4)
Platelet ($x10^4/mm^3$)	20.3 (13.7-26.8)	23.2 (13.4-40.5)	21.0 (11.9-30.5)	12.0 (3.9-25.1)
Albumin (g/dL)	4.6 (4.1-4.8)	4.5 (3.9-5.4)	4.4 (3.5-4.9)	3.8 (2.6-4.6)
Total bilirubin (mg/dl)	0.7 (0.4-1.6)	0.8 (0.3-2.4)	0.9 (0.4-2.4)	1.3 (0.3-2.5)
AST (IU/U)	32 (19-45)	54(17-245)	80 (31-186)	52 (27-120)
ALT (IU/L)	56 (14-102)	96 (12-539)	116 (43-318)	50 (14-138)
Type IV collagen 7s (ng/ml)	4.2 (3.2-5.2)	5.0 (2.7-8.1)	6.1 (3.8-8.9)	7.7 (6.7-8.6)
Hyaluronic acid (ng/ml)	43 (12-92)	115 (12-751)	82 (13-231)	192 (29-451)
APRI	0.50 (0.27-0.91)	0.72 (0.25-2.86)	1.24 (0.58-3.41)	1.69 (0.44-3.57)
FIB-4 index	1.52(0.46-3.24)	1.53 (0.55-3.08)	2.44 (0.70-3.94)	6.57 (1.36-15.89)
Steatosis (0/1/2/3)	1/2/3/6	0/8/5/8	0/11/7/5	1/4/3/1
Hepatocyte Balloning (0/1/2)	3/9/0	1/11/9	0/10/13	0/5/4
Lobular Inflammation (0/1/2/3)	0/9/3/0	0/13/7/1	0/9/12/2	1/4/3/1

Supplementary Table S2. Clinical Background of Chronic Hepatitis for HCV Patients in Each Stage of Fibrosis

	Chronic hepatitis with HCV				
	Stage 0-1 (n = 18)	Stage 2 (n= 12)	Stage3 (n = 3)	Stage4 (n=15)	HCC (n=31)
Male/Female	9/9	7/5	1/2	4/11	19/12
Age (year)	52 (18-27)	65 (49-74)	65 (54-76)	67 (46-89)	72 (58-85)
Platelet ($x10^4/mm^3$)	18.1 (13.0-34.1)	15.7 (8.3-21.4)	8.8 (8.1-11.6)	10.0 (3.5-16.9)	9.1 (3.4-20.7)
Albumin (g/dL)	4.4 (3.8-4.9)	4.1 (2.9-4.6)	3.8 (3.5-4.4)	3.7 (2.5-4.1)	3.5 (1.9-4.4)
Total bilirubin (mg/dl)	0.6 (0.4-1.3)	0.8 (0.4-1.1)	0.8 (0.7-1.5)	0.9 (0.6-4.7)	0.8 (0.3-16.7)
AST (IU/U)	31 (17-99)	51 (12-29)	71 (37-206)	56 (42-110)	46 (21-315)
ALT (IU/L)	35 (17-176)	55 (18-116)	84 (31-294)	47 (21-93)	39 (13-160)
Type IV collagen 7s (ng/ml)	3.9 (2.3-5)	5.7 (4-9.8)	7.4 (6.3-8.5)	11 (7.6-16.0)	8.9 (4.3-225.0)
APRI	0.50 (0.2-2.0)	0.85 (0.4-2.2)	2.4 (1.4-5.4)	2.2 (1.3-9.5)	1.41 (0.6-10.5)
FIB-4 index	1.64 (0.55-2.79)	2.62 (1.15-6.42)	5.59 (5.33-6.69)	6.59 (3.69-22.0)	5.73 (2.63-23.45)
Histological Activity (0/1/2/3)	0/15/3/0	0/3/9/0	0/0/3/1	0/7/7/1	
AFP (ng/ml)					17.5 (3-1859)
PIVKA-II (mAU/ml)					33 (8-3560)

Data are presented as the median values (range)

BMI, APRI see Table1

Supplementary Figure Legends

Supplementary Figure S1: Type IV collagen 7s, hyaluronic acid, WFA$^+$ -M2BP, APRI, FIB-4 index in patients with NAFLD with different stages of fibrosis.

The serum levels of type IV collagen 7s (a), hyaluronic acid (b), and WFA$^+$ -M2BP (c) APRI (d), and FIB-4 index (e) are shown. *, p<0.05; **, p<0.001 Kruskal-Wallis test with Dunn's multiple comparison test. The stage of fibrosis was assessed according to Brunt classification.

Supplementary Figure S2: **Serum levels of YKL-40 in patients with HCV infection with different stages of fibrosis**.

Serum YKL-40 levels are shown in patients with HCV stratified by the stage of fibrosis (METAVIR classification). * p<0.05, ** p<0.001, by Kruskal-Wallis test with Dunn's multiple comparison test.

HV, healthy volunteers

Supplementary Figure S3: ROC analyses of serum YKL-40, other markers and fibrosis scores for the diagnosis of advanced fibrosis in patients with NAFLD.

This figure shows ROC curves of YKL-40, type IV collagen 7s, hyaluronic acid, WFA$^+$ -M2BP, APRI, FIB-4 index, and the prediction model for NAFLD established by YKL-40 and type IV collagen 7s as a diagnostic marker of advanced fibrosis (Stage 3-4 vs. Stage 0-2).

Supplementary Figure S4: Expression and production of YKL-40 from macrophages in vitro.

Primary macrophages were generated from monocytes obtained from healthy donors as described in Materials and Methods. a: The mRNA levels of YKL-40 in M-CSF-differentiated macrophages were examined on day 5, 6 and 7 of the culture. b: The supernatants were collected from day 7 macrophages stimulated with (conc.) TNF-α and/or (conc.) IL-1β. The YKL-40 levels in the supernatants were assayed by ELISA. * $p<0.05$, ** $p<0.001$ by Student t-test.

Supplementary Figure S5: **Serum YKL-40 levels were not correlated with serum TNFα levels and serum IL-1β levels.**

The correlations between serum YKL-40 levels and serum TNFα or IL-1β levels were assessed by Spearman's rank-correlation coefficient. The p-values and correlation coefficients are depicted in each plot.

Supplementary Figure S6: **Serum YKL-40 levels in CHC patients with or without HCC.**

Serum YKL-40 levels in chronic hepatitis and liver cirrhosis patients with or without HCC are shown. * $p<0.05$, by Kruskal-Wallis test with Dunn's multiple comparison test

Supplementary Figure S1

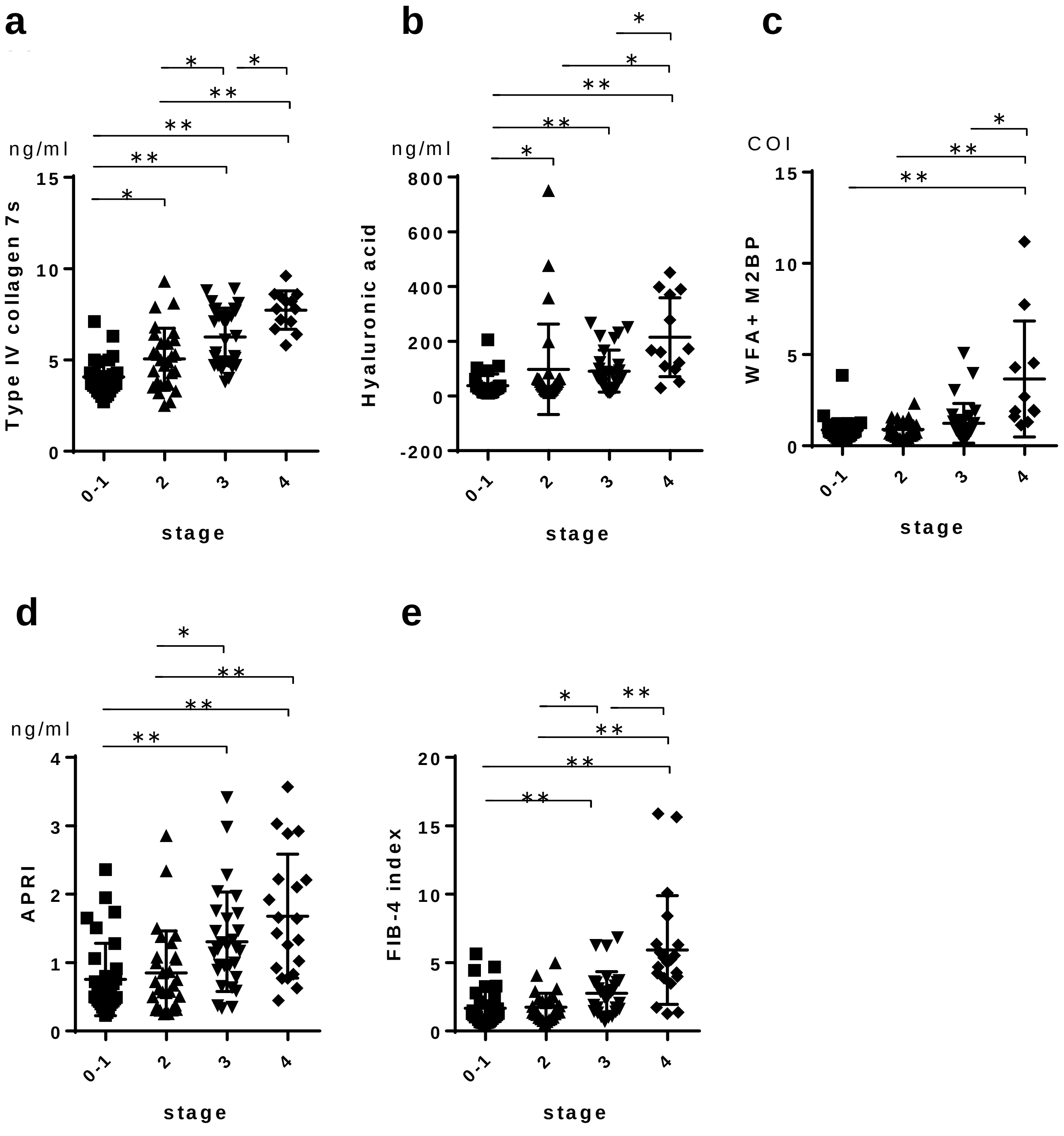

Supplementary Figure S2

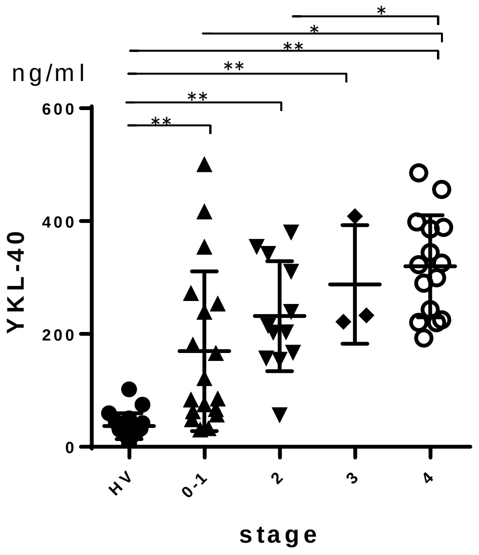

Supplementary Figure S3

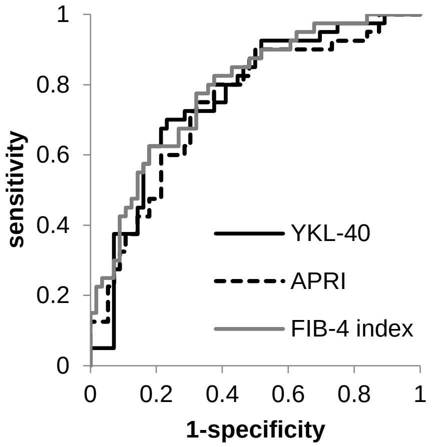

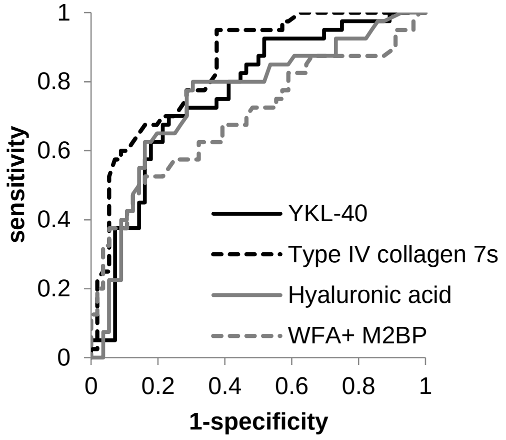

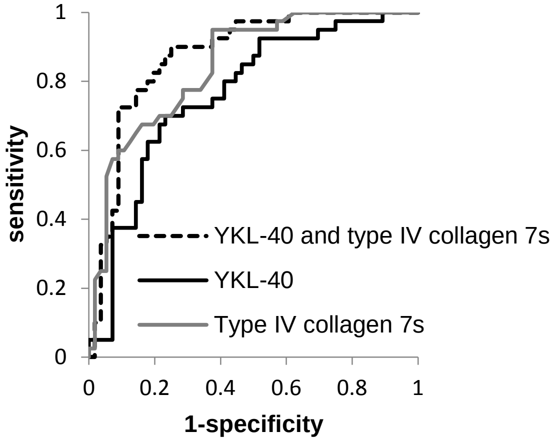

Supplementary Figure S4

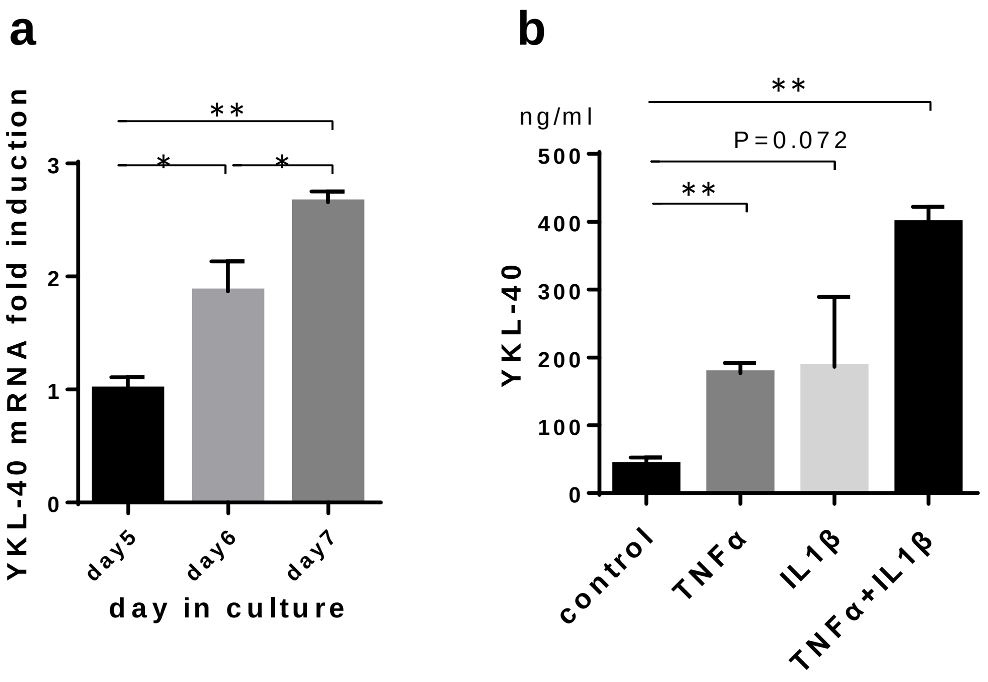

Supplementary Figure S5

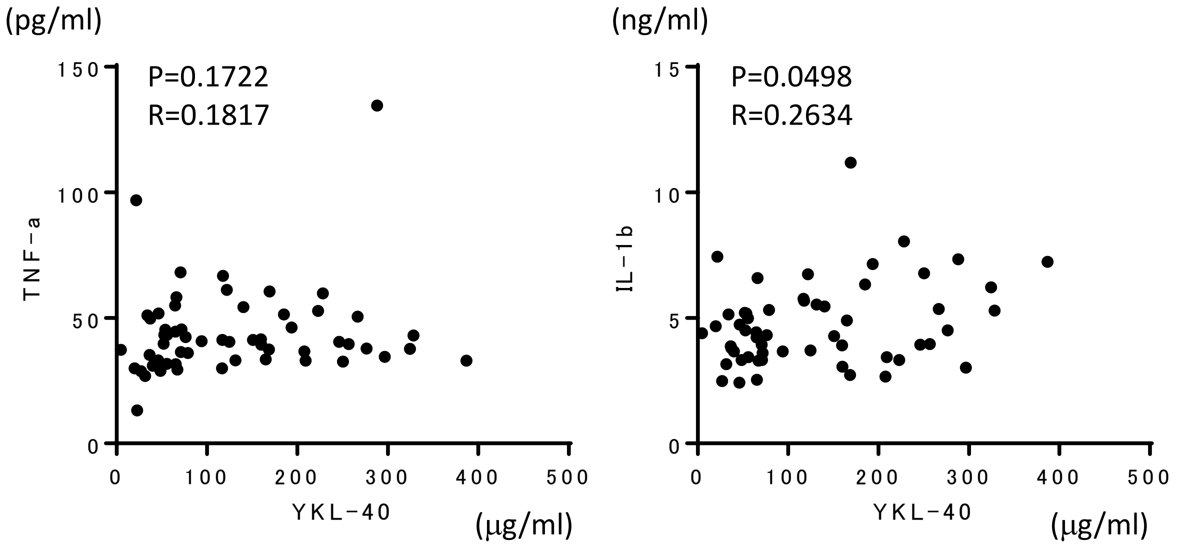

Supplementary Figure S6

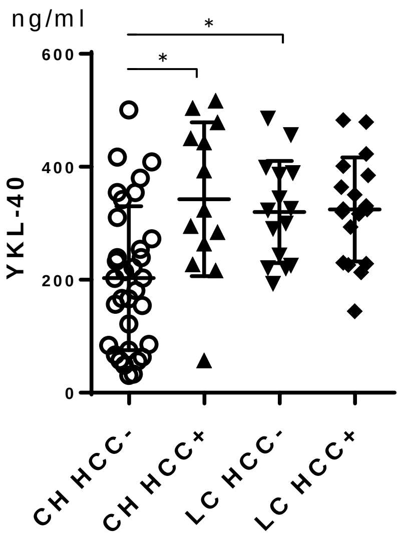

Supplement: Supplementary Information [file srep35282-s1.pdf]
